# Re-evaluation of a 2014 multi-country European outbreak of *Salmonella* Enteritidis phage type 14b using recent epidemiological and molecular data

**DOI:** 10.2807/1560-7917.ES.2017.22.50.17-00196

**Published:** 2017-12-14

**Authors:** Stefan Hörmansdorfer, Ute Messelhäußer, Albert Rampp, Katharina Schönberger, Tim Dallman, Franz Allerberger, Christian Kornschober, Andreas Sing, Peter Wallner, Andreas Zapf

**Affiliations:** 1 Bavarian Health and Food Safety Authority, Oberschleißheim, Germany; 2 Gastrointestinal Bacterial Reference Unit, London, United Kingdom; 3 Austrian Agency for Health and Food Safety (AGES), NRC Salmonella, Graz, Austria

**Keywords:** Salmonellosis, *Salmonella* Enteritidis PT14b, multi-country outbreak, food-borne infections, egg consumption, Whole Genome Sequencing, WGS

## Abstract

A European multi-country outbreak of *Salmonella* Enteritidis phage type (PT) 14b occurred from March to November 2014 associated with the consumption of eggs. The outbreak involved more than 400 human cases from France, Luxembourg, Austria and the United Kingdom. In 2016–2017, it has been re-evaluated combining recent epidemiological results with latest molecular data. The outbreak was traced back to one large Bavarian egg producer with four distinct premises, three located in Bavaria, one in the Czech Republic. The outbreak isolates of *S.* Enteritidis PT 14b were grouped into three closely related clades by whole genome sequencing. Two of these clades could be referred to two Bavarian premises of the egg producer on the basis of epidemiological and molecular data, while epidemiological data presumably linked the third clade to another premises of the egg producer. Interestingly and in contrast to the situation in other European countries where several outbreaks were documented, all notified 91 laboratory-confirmed cases of *S.* Enteritidis PT 14b from Bavaria were sporadic, singular cases not belonging to any epidemiological outbreaks. In conclusion, as demonstrated here, the resolution of food-related outbreaks with such a high discriminatory power is rare in outbreak investigation.

## Introduction

*Salmonella enterica* ssp. *enterica* serovar Enteritidis (*S.* Enteritidis) is one of over 2,600 serovars of the genus *Salmonella* with zoonotic potential and often associated with food-borne human disease. Its reservoir is the intestinal tract of both humans and animals. *S.* Enteritidis forms a strongly clonal group with low genetic heterogeneity, whose members can be sufficiently discriminated from each other only by modern molecular techniques [1-6].

The incidence of human salmonellosis has decreased steadily in recent years. Nevertheless, in 2014, 88,175 confirmed cases of human salmonellosis causing 9,830 hospitalisations and 65 fatalities were reported across the European Union (EU). Among these, 16,000 cases of human salmonellosis were reported from Germany. As in previous years, *S.* Enteritidis was the predominant serovar (44.4% of all isolates) followed by *S.* Typhimurium (17.4%) and *S.* Typhimurium, monophasic variant (7.8%) [7].

Poultry meat and eggs have been shown as being potentially contaminated by *Salmonellae* [8,9] and consumers should be fully aware of that [10]. The EU has prescribed community targets for the reduction of *S.* Enteritidis and *S.* Typhimurium with Regulation (EC) 1168/2006, in adult laying hens of *Gallus gallus*, which are monitored by mandatory control programmes since 2009 [11]. These programmes have contributed to the decline of *Salmonella*-contaminated flocks of laying hens [2,12]. Only 2.54% of 34,757 flocks of laying hens were *Salmonella*-positive within the EU in 2014. *S.* Enteritidis was detected in 0.7% of all flocks. In Germany, only 1.39% of 5,256 flocks of laying hens were *Salmonella*-positive with *S.* Enteritidis detected in 0.44% of them [7]. Although *Salmonella* spp. in table egg units are only rarely detected (0.3% of single samples and 1% of batches in 2014), eggs and egg products were important sources of food-related *Salmonella* outbreaks in 2014 [5]. Phage type (PT) 14b of *S.* Enteritidis was reported in several food-related outbreaks in recent years, often with eggs or egg products as suspected or proven source [13-15].

## The outbreak

From March to November 2014, a European multi-country outbreak occurred in France, Luxembourg, Austria and the United Kingdom (UK) with more than 400 human cases. The outbreak strain was at that time identified as *S.* Enteritidis, PT 14b, multilocus variable-number tandem repeat analysis (MLVA) type 2–12–7-3–2 or 2–11–7-3–2 [1,5,16,17]. The outbreak was epidemiologically traced back to eggs from one large egg producer (company X) with three premises A, B and C in Bavaria and premises D in the Czech Republic. Besides that, company X owns two breeding units, one in Bavaria and one in the Czech Republic.

Following a Rapid Alert System for Food and Feed within the EU (RASFF) notification about a *S.* Enteritidis outbreak in France after consuming homemade ice cream prepared with raw eggs traced back to company X in early July 2014 [18], Bavarian authorities initiated extensive and integrated investigations at the three Bavarian premises A, B and C of company X. *S.* Enteritidis was cultured from eggs (surfaces and contents) and from faeces and stable dust at premises A and from egg surfaces at premises B. *S.* Enteritidis was not detected in the respective samples or materials obtained at the third Bavarian premises C. Here we report the re-evaluation of this multi-country European outbreak of *S.* Enteritidis PT 14b by combining recent epidemiological results with latest molecular data.

## Methods

For the re-evaluation we used all relevant RASFF notifications and posts on the European Centre for Disease Prevention and Control (ECDC)’s Epidemic Intelligence Information System (EPIS), epidemiological outbreak reports from ECDC / European Food Safety Authority (EFSA) [16] and from the concerned Austrian authorities [17] and we assessed and analysed published results of the UK outbreak investigation [5]. Moreover, data obtained from investigations performed by local Bavarian Health authorities together with the analysis of questionnaires from Bavarian cases as well as bilateral epidemiological information obtained directly from Austrian authorities [19] were included.

In a next step, we compared the obtained epidemiological data with the latest molecular results from whole genome sequencing (WGS) of European isolates, performed by Public Health England (PHE) [1]. All UK outbreak strains and further 45 isolates from France, Luxembourg, Austria and Germany were analysed by WGS. Bavarian environmental isolates and isolates from eggs of premises A and B of company X, which were obtained during official investigations, were also included.

## Results

### France

Six food-related outbreaks caused by homemade food prepared with raw eggs involving 45 human cases were reported in France between June and July 2014. *S.* Enteritidis was isolated from 16 human cases and from one egg sample taken in a private kitchen. The outbreaks were traced back epidemiologically to premises A and B of company X [16].

### Luxembourg

Luxembourg reported one human case infected with the outbreak strain in early June 2014. The patient residing in France close to the border to Luxembourg, consulted a hospital in Luxembourg and had consumed eggs possibly bought in a shop of the supermarket chain linked to the French cases [16].

### Austria

Between June and October 2014, 151 *S.* Enteritidis PT 14b cases (90 confirmed, 38 probable and 23 possible cases) in Austria fulfilling the Austrian case definition, occurred with an accumulation of 69 cases in Tyrol as at 22 October 2014 [17,19]. A confirmed case was defined as an infection with *S.* Enteritidis PT14b (MLVA-type 2–12–7-3–2) occurring after week 23 (starting on 2 June) 2014 in a person living in Austria. A probable case was defined as an infection with *S.* Enteritidis PT14b without MLVA-typing occurring after week 23 in a person living in Austria. A possible case was defined as diarrhoea or vomiting occurring after week 23 in a person living in Austria and having an epidemiological link to a confirmed case [17].

In 51 of 69 Tyrolian cases (74% in total; 16 confirmed, 1 probable and 34 possible cases) the consumption of partly raw eggs-containing food was identified as common exposure. The food was prepared in a large-scale catering facility at Innsbruck. The food was delivered to ‘meals on wheels’ customers and residents of three nursing homes for the elderly, which were supplied by the above mentioned caterer. The large-scale catering facility bought eggs via a distributor in Innsbruck from an egg selling company in Bavaria which was supplied by company X. The Austrian authorities sampled 10 eggs delivered from premises B of company X on 4 July 2014 as well as 10 further eggs, beef soup powder, salt, corn starch, four different spices and one counter sample (vegetable casserole) from the large scale catering facility on 9 July 2014. *Salmonella* spp. could neither be isolated from the food nor from the egg samples. During the outbreak period, a member of the kitchen staff, a food distributor, was identified as carrying the outbreak strain [17,19]. They were probably infected by contaminated eggs or egg containing food.

### United Kingdom

The UK reported an outbreak with a duration of 17 weeks from May to November 2014 [1]. Besides 101 sporadic cases there were five outbreaks with 259 cases, linked to a hospital, three Chinese restaurants and one kebab restaurant [1] and (personal communication, Tim Dallman, February 2017). Sixty-nine per cent (198/287) of confirmed cases could be epidemiologically linked to eggs supplied by company X [5]. *S.* Enteritidis PT 14b with the outbreak MLVA profile was isolated from environmental and food samples from the hospital and two restaurants, but not from eggs [5].

### Germany

In Germany, 177 *S.* Enteritidis PT 14b cases were identified by phage typing in 2014 at the National Reference Centre (NRC) for Salmonella and other Bacterial Enteric Pathogens, Wernigerode, of which 107 were from Bavaria (personal communication, Wolfgang Rabsch, October 2016). Importantly, it should be noted that phage typing is not routinely performed in Germany. Only those isolates sent to the National Reference Centre for Salmonella and other Bacterial Enteric Pathogens are phage typed. Thus, the occurrence of different phage types among German *Salmonella* isolates is generally underestimated. The Bavarian Health and Food Safety Authority initiated phage typing since July 2014 for the Bavarian regions Upper Palatine and Lower Bavaria and since August 2014 for all Bavarian regions. Since August 2014, the national public health institute (Robert Koch Institute, Berlin) initiated phage typing for the other federal states on a voluntary basis. In Table 1 the number of cases and their incidence per 100,000 inhabitants in different Federal States of Germany in 2014 are described. Most of the cases and the highest incidence were observed in Bavaria, reflecting the intensified surveillance in Bavaria since July 2014. Ten or more *S.* Enteritidis PT 14b cases were observed in each of the federal states of Saxony, North Rhine-Westphalia and Lower Saxony.

**Table 1:** Cases of *Salmonella* Enteritidis phage type 14b, Germany, 2014

Federal state of Germany	Number of cases	Inhabitants	Incidence per 100,000 inhabitants
Baden-Württemberg	6	10,879,618	0.06
Bavaria	107	12,843,514	0.83
Berlin	0	3,520,031	NA
Brandenburg	1	2,484,826	0.04
Bremen	1	671,489	0.15
Hamburg	3	1,787,408	0.17
Hesse	7	6,176,172	0.11
Lower Saxony	10	7,926,599	0.13
Mecklenburg-West Pomerania	1	1,612,362	0.06
North Rhine-Westphalia	12	17,865,516	0.07
Rhineland Palatinate	2	4,052,803	0.05
Saarland	0	995,597	NA
Saxony	17	4,084,851	0.42
Saxony-Anhalt	4	2,245,470	0.18
Schleswig Holstein	3	2,858,714	0.10
Thuringia	3	2,170,714	0.14
Total	177	82,175,684	0.22

NA: not applicable.

Source: Database of the National Reference Centre for Salmonella and other Bacterial Enteric Pathogens, Wernigerode, personal communication, Wolfgang Rabsch, October 2016 and [20].

In 2014, 91 Bavarian cases of Salmonella Enteritidis PT 14b were notified according to the German Infection Protection Act (SurvNet-Database of RKI, as at 31 March 2015). Ten of them were asymptomatic carriers, among whom one was an employee of company X. All Bavarian cases were sporadic, singular cases that did not belong to any epidemiological cluster or outbreak. All of them were asked to take part in an in depth questionnaire-based interview with a healthcare official on voluntary basis. Just over half of the cases (55%; n = 50) agreed to be interviewed. According to the questionnaire responses, 19 cases consumed eggs, but five of them had acquired their *Salmonella* infection abroad. The consumption of raw egg-containing food could be excluded for four persons. For the remaining 10 cases the origins of the consumed eggs were: unknown (n = 4), supermarket without further details (n= 2), eggs from free-range hens (n = 1), organic eggs without further details (n = 1), eggs directly bought at the farm (n = 1) and eggs from one’s own hens (n = 1). Taken together, no link to consumption of eggs of company X practicing cage production could be identified by these interviews.

### Molecular investigation by whole genome sequencing

The outbreak strains formed a single five single nucleotide polymorphism (SNP) single-linkage cluster with a maximum distance between any two genomes of 23 SNPs. Three clades could be separated within the monophyletic cluster with a maximum difference of two SNPs [1]. The corresponding phylogenetic tree is published elsewhere [1]. The time to the most recent common ancestor for the three clades was estimated to be 2.9 years (95% highest posterior density (HPD): 2.5–3.2 years) [1]. The analyses referred all three clades to company X or its egg supply network and postulated a common ancestor of all three clades [1].

All sequenced European strains can be assigned to the three clades as shown in Table 2 with the exception of two human strains from France and one human strain from Germany which could not be assigned to any of the three clades [1].

**Table 2:** Assignment of the European outbreak strains of *Salmonella* Enteritidis phage type 14b to three different clades and to single premises of company X

**Clade 1, isolates from faeces, dust samples and eggs, directly sampled at premises A of company X (n=10)**			
**Country**	**Outbreak**	**Origin of isolates** **(number)**	**Comment**
France	Outbreak after consumption of chocolate cream produced with raw eggs (RASFF-notification 2014.0938-inf01) Outbreak after consumption of ice cream produced with raw eggs (RASFF-notification 2014.0938-inf01)	Eggs (n=2) Human (n=2)	Epidemiological link to company X Epidemiological link to company X
United Kingdom	Sporadic cases	Human (n=3)	NA
Germany	Sporadic cases	Human (n=3)	No epidemiological link to company X
**Clade 2 Isolates from eggs, directly sampled at premises B of company X (n=2)**			
**Country**	**Outbreak**	**Origin of isolates** **(number)**	**Comment**
France	Outbreak after consumption of mayonnaise produced with raw eggs (RASFF-notification 2014.1072)	Human (n=3)	Epidemiological link to company X
	Sporadic cases	Human (n=2)	No epidemiological link to company X
Luxembourg	Sporadic case	Human (n=1)	Epidemiological link to company X
Austria	Outbreak in Tyrol in June/July 2014, caused by a caterer delivering food at homes for the elderly and “meals on wheels” to risk groups	Human (n=3)	Epidemiological link to company X through egg supply network
	Human isolates not belonging to the Tyrolian outbreak	Human (n=2)	Isolates from Upper Austria and Vienna^a^
United Kingdom	Outbreak in a kebab house	Human (n=9)	Epidemiological link to company X
	Sporadic cases	Human (n=23)	NA
Germany	Sporadic cases	Human (n=7)	No epidemiological link to company X
	Sporadic cases	Human (n=1)	Epidemiological link to company X (employee of company X, asymptomatic carrier)
**Clade 3, no isolate from any premises of company X**			
**Country**	**Outbreak**	**Origin of isolates** **(number)**	**Comment**
Austria	Human isolate not belonging to the Tyrolian outbreak	Human (n=1)	Isolate from Vienna^a^
	Sporadic cases	Human (n=1)	Isolate was cultured in 2013 and has no epidemiological link to the outbreak in 2014^a^
Germany	Sporadic cases	Human (n=4)	No epidemiological link to company X
United Kingdom (UK)	Outbreaks in three Chinese restaurants and a UK hospital	Human (n=250)	Epidemiological link to company X
	Sporadic cases	Human (n=75)	NA

RASFF: Rapid Alert System for Food and Feed.

^a ^Source: personal communication: Tim Dallman, February 2016.

Source: [1] and personal communication: Wolfgang Rabsch, January 2017; Tim Dallman, February 2017.

### Combination of epidemiological and molecular results

The synoptic view of all epidemiological data with molecular typing data from PHE yielded the following scenario of the 2014 multi-country outbreak with epidemiological data confirming molecular results (Table 2).

Isolates of clade 1 were linked to premises A of company X based on molecular analysis of isolates from premises A. Two outbreaks in France were linked to premises A of company X due to molecular analysis of isolates from humans and eggs and the identification of premises A of company X as supplier of the eggs. Interventions at premises A, i.e. removal of the old flock between 20 June and 22 July 2014, immediate housing of a new flock, which never supplied table eggs, coincided with the cessation of the appearance of clade 1 isolates after the end of July 2014.

Isolates of clade 2 were linked to premises B of company X based on molecular analysis of isolates from premises B. One outbreak in France and one outbreak in a kebab house in the UK could be related to premises B of company X. Human isolates from France were linked to clade 2 by molecular analysis, while premises B of company X was identified as egg supplier. Isolates from the UK were traced back epidemiologically to company X through the egg supply network. After a delivery stop for eggs from premises B of company X to the United Kingdom on 17 July 2014 and the passing of the eggs’ shelf life only a few singular isolates of clade 2 were detected in the UK.

Isolates of clade 2 and clade 3 were cultured from Austrian outbreak cases. Seven Austrian isolates were sequenced by WGS. Five of them belonged to clade 2, two of them to clade 3. One of the clade 3 isolates was cultured in 2013 (personal communication, Tim Dallman, February 2016) and could not be attributed to the 2014 outbreak.

Most of the UK cases could be linked to company X due to the egg supply network (Table 2) as reported elsewhere [5]. The major part of the UK isolates including the isolates from four of five outbreaks in the UK (one hospital and three Chinese restaurant outbreaks) belongs to clade 3. Isolates of clade 3 were continuously detected in the UK from March to November 2014. Interventions at premises A and B of company X did not influence the appearance of clade 3 isolates. However, after a delivery stop for eggs of premises D of company X from the Czech Republic to the UK on 1 September 2014 and the passing of the eggs’ shelf life, only singular, sporadic cases of clade 3 isolates without further outbreaks were detected in the UK. Sixteen samples from premises D of company X (faeces (n=4), whole body (n=6), container (n=6)) were analysed by the Czech authorities between 3 March 2014 and 10 July 2014. *Salmonella* spp. were not detected as reported by the Czech government via the RASFF system.

## Discussion and conclusions

Re-evaluating this large multi-country outbreak by combining the results of recent molecular and epidemiological data allowed us to clearly attribute some outbreaks to single premises of one great egg supplier, company X. The French outbreaks could be referred to premises A and B and the UK outbreak in a kebab house to premises B of company X.

In Austria, clade 2 and clade 3 strains were isolated during the outbreak. Although the isolation of *S.* Enteritidis from eggs or incriminated food failed, it can be supposed that premises B of company X was involved in the Tyrolian outbreak due to an epidemiological link between the large-scale catering facility at Innsbruck and company X via the egg supply network and the isolation of clade 2 strains from clinical cases. It is notable that 18 cases infected with a strain of S. Enteritidis PT 14b, indistinguishable from the outbreak strain 2014 by MLVA typing were detected both in Tyrol and other federal states of Austria already in October and November 2013 [17].

The appearance of clade 3 strains in Austria before and during the outbreak gives a hint that a further, yet unknown source might have been involved. For a more exact characterisation of the Austrian outbreak the sequencing of a greater number of Austrian outbreak strains would have been helpful. The fact that a human carrier of the outbreak strain worked as food distributor at the caterer as well as the distribution of raw egg containing food may have influenced the extent and the duration of the Austrian outbreak.

The source of clade 3 strains which were isolated from most of the UK cases remains obscure. There is the possibility that *S.* Enteritidis was merely not detected from eggs or environmental samples from premises C of company X in Bavaria, despite intensive sampling. In addition, the molecular relationship of all UK outbreak strains provides some epidemiological evidence that the source for clade 3 strains could be premises D of company X in the Czech Republic. This hypothesis is supported by the fact that UK clade 3 cases strongly decreased and only few sporadic cases were detected after the delivery stop on 1 September 2014 for eggs from the Czech premises D of company X and after the expiration of their shelf life.

As molecular data postulate a common ancestor strain of all three clades ca 2.9 years ago, we suppose that the ancestor strain was introduced from an external parent or grandparent herd to the premises of company X in the past and started to develop independently at each premises finally forming the three clades. This hypothesis is supported by the fact that *S.* Enteritidis PT 14b clade 2 strains were isolated during a *S.* Enteritidis hospital outbreak in Bavaria in summer 2015 and from the laying hens of the hospital’s egg supplier. In this outbreak *S.* Enteritidis PT 14b clade 2 was isolated from 11 persons who were directly or indirectly linked to a Bavarian hospital in Swabia (patients (n=6), relatives (n=2), trainee (n=1), cook (n=1) and cook mate (n=1)). The route of transmission inside the hospital could not be clarified, but it was supposed that *S.* Enteritidis was transmitted via cross-contaminated food from the kitchen. The most likely primary source was identified as eggs from a local egg supplier about two hundred kilometres away from the premises of company X (data not shown).

An official sampling of the five laying hen herds (separate faecal/dust samples from every herd and 120 eggs from the egg packing centre) of the egg supplier was done and *S.* Enteritidis PT 14b clade 2 was isolated from one of the five laying hen herds (faecal/dust samples). The egg samples taken in the egg packing centre of the company were negative for *Salmonella* spp. In a routine self-check of the egg supplier done incidentally one week before the official sampling, *S.* Enteritidis was also isolated from a second laying hen herd. Summarising the results, two of the five laying hen herds of the local egg supplier tested positive for *S.* Enteritidis PT 14b clade 2 (data not shown).

In contrast to company X, the egg supplier received *Salmonella*-immunised young hens from a local breeding farm in the north of Bavaria and kept them in deep litter. A connection between the local egg supplier and company X (e. g. feed, water supply, litter) could not be identified. Besides, the whole flock of premises B of company X shedding clade 2 strains had been slaughtered in September 2014, 11 months before the hospital outbreak.

### Conclusions

Comprehensive outbreak investigations require the combination of epidemiological data and molecular typing techniques, especially if bacteria with a very low natural mutation rate such as *Salmonella* spp. are concerned. Low discriminatory typing techniques like phage typing or MLVA typing may be well suited for investigating small, circumscribed, local outbreaks. However, the investigation of large-scale, long-lasting, cross-border outbreaks calls for highly discriminatory techniques such as WGS to allow distinct and unambiguous assignment and classification of isolates.

The collection of epidemiological data from different sources together with molecular typing data enabled us to retrospectively evaluate the 2014 multi-country *S.* Enteritidis PT 14b outbreak tracing back the incriminated food across country borders to its source at the farm. We were even able to link different parts of the outbreak to distinct premises of one large egg supplier. A resolution of food-related outbreaks with such a high discrimination is a very rare result in outbreak investigations. The close collaboration of the participating authorities of all countries was crucial in achieving this.
